# Physical Activity, Academic Stress, and Motivation in Physical Education: A Cross-Sectional Analysis by Sex and Grade Level

**DOI:** 10.3390/healthcare14142158

**Published:** 2026-07-17

**Authors:** Borys Bismark León-Reyes, Dilan Galeano-Rojas, Marcos Granados-Moreno, Josivaldo de Souza-Lima, Claudio Farías-Valenzuela, Antonio Castillo-Paredes, Pedro Valdivia-Moral

**Affiliations:** 1 Facultad de Educación, Universidad Estatal de Milagro, Milagro 091050, Ecuador; bleonr@unemi.edu.ec; 2 Departamento de Didáctica de la Expresión Musical, Plástica y Corporal, Facultad de Ciencias de la Educación, Universidad de Granada, 18071 Granada, Spain; dagaleanor@correo.ugr.es (D.G.-R.); marcosgranados@correo.ugr.es (M.G.-M.); 3 Instituto del Deporte y Bienestar, Facultad de Educación y Ciencias Sociales, Universidad Andres Bello, Las Condes, Santiago 7591538, Chile; josivaldo.desouza@unab.cl; 4 Escuela de Ciencias de la Actividad Física, el Deporte y la Salud, Universidad de Santiago de Chile (USACH), Santiago 9170022, Chile; claudio.farias.v@usach.cl; 5 Grupo AFySE, Investigación en Actividad Física y Salud Escolar, Escuela de Pedagogía en Educación Física, Facultad de Educación, Universidad de Las Américas, Santiago 8370040, Chile; acastillop85@gmail.com

**Keywords:** academic stress, physical activity, motivation, physical education

## Abstract

**Objectives**: The aim of the present study was to analyze the relationships among physical activity, academic stress, and motivation in Physical Education, as well as to determine differences according to sex and grade level. **Methods**: A total of 310 secondary education students participated in the study. Data were collected using the PAQ-C, the Questionnaire on Academic Stress in Secondary Education, and the Motivation in Physical Education Questionnaire. Spearman’s correlation test and a MANOVA were used for the statistical analysis. **Results**: The findings revealed that physical activity was positively correlated with more self-determined forms of motivation and negatively correlated with amotivation. The dimensions of academic stress were positively associated with amotivation. Regarding sex differences, statistically significant differences were found in physical activity, with higher levels among males, and in academic overload, with higher values among females. According to grade level, differences were identified in academic overload, intrinsic motivation, and identified regulation. Furthermore, the sex × grade interaction was also statistically significant, showing that females reported higher levels of stress as they progressed through educational stages, whereas in males, levels of academic stress decreased and both physical activity and intrinsic motivation showed a differential increase in the final year of secondary education. **Conclusions**: In this regard, it is concluded that sex and grade level influence the variables analyzed. However, further research is needed to better understand these dimensions, their impact, and their interrelationships, considering the potential benefits of promoting self-determined motivation in Physical Education and encouraging regular physical activity practice, particularly in reducing and coping with academic stress, with the aim of comprehensively improving the physical and mental well-being of the adolescent population and contributing to their healthy and balanced development.

## 1. Introduction

Physical Education (PE) plays an essential role in promoting physical and mental health, as well as in fostering healthy lifestyle habits that may persist into adulthood [1,2,3,4]. From a holistic perspective, PE encompasses affective, cognitive, psychomotor, and social dimensions, constituting a key setting for promoting well-being and preventing public health issues, including physical and psychological deterioration [5]. However, within the context of secondary education—a stage during which students experience significant physical, psychological, and social changes—it is essential to understand how participation in PE may influence their ability to cope with the challenges inherent to this developmental period [6].

Several studies suggest that participation in PE can improve social skills and mental health, reducing vulnerability to disorders such as stress, anxiety, and depression [3,7]. Stress, defined as an adaptive response to environmental demands that exceed an individual’s resources [8], is a prevalent phenomenon in the school context, affecting adolescents’ behavior and well-being [9,10]. The literature indicates that academic stress represents one of the main sources of psychological distress among this population and is associated with low self-esteem, difficulties in self-regulation, and anxiety and depressive disorders [11,12,13,14]. These issues not only affect mental health, but also academic performance and school participation [15,16].

In this regard, Physical Activity (PA) has been identified as a coping mechanism capable of modulating the impact of stress and promoting psychological well-being [17,18]. The World Health Organization (WHO) [19] defines PA as any bodily movement produced by skeletal muscles that requires energy expenditure and that, at moderate and vigorous intensities, improves physical and mental health. Furthermore, the WHO recommends that children and adolescents aged 5 to 17 years engage in at least 60 min of moderate-to-vigorous PA daily; however, most adolescents do not meet these recommendations, particularly girls. Evidence demonstrates that regular participation in PA contributes to reducing levels of anxiety, depression, and stress while also improving self-confidence, self-esteem, and social relationships [18,20,21,22,23,24]. From a physiological perspective, PA helps regulate the stress response by reducing cortisol levels and stimulating the release of endorphins, generating feelings of well-being and pleasure that promote emotional resilience and academic performance [25,26,27].

Nevertheless, despite the potential of PA and PE in promoting well-being, several reports indicate that participation rates in PA among adolescents remain insufficient, with more than 80% of young people failing to meet the minimum recommendations. This situation is exacerbated by gender differences and the increasing prevalence of mental health problems and academic stress [19,20,21,22,23,24,25,26,27,28]. Self-Determination Theory [29] posits that motivation (intrinsic–extrinsic) is a key factor in promoting sustained participation in PA. Intrinsically motivated students participate in class due to the satisfaction and enjoyment derived from the activity itself, whereas extrinsically motivated students tend to focus on the outcomes and rewards they may obtain, or even on avoiding negative consequences [30]. It has been demonstrated that the satisfaction of basic psychological needs—autonomy, competence, and relatedness—enhances well-being and may increase engagement in physical activities, as well as strengthening coping mechanisms against stress [31]. However, despite the existing evidence linking motivation, PA, and stress among adolescents, there remains a gap in understanding how these variables interact within the school context, particularly regarding PE practices and their potential to mitigate academic stress.

Various factors influence low levels of motivation toward PA, including family support, social networks, teaching style, infrastructural conditions, academic overload, and assessment strategies [2,3,32,33]. These factors contribute not only to reduced participation in PE, but also to the persistence of psychological distress and academic stress, generating a cycle that may negatively affect mental health and academic performance and even lead to extracurricular sports dropout [26,34,35].

Therefore, the present study seeks to contribute to this conceptual gap by proposing a cross-sectional analysis of the relationships among PA, academic stress, and motivation in secondary education students, with the aim of better understanding how these factors interact and how motivation in PE may act as a coping mechanism against academic stress, thereby contributing to the promotion of active and sustained participation in physical activities among this population. In this sense, the objectives of this study are: (i) to analyze the relationships among PA, academic stress, and motivation in PE among secondary school students and (ii) to determine differences in these variables according to sex and academic grade level. Accordingly, the following study hypotheses are proposed:

**H1.**  *More self-determined forms of motivation will be associated with higher levels of PA and lower academic stress.*

**H2.**  *Higher levels of PA will be associated with lower academic stress.*

**H3.**  *Statistically significant differences will be found in these variables according to sex, grade level, and the interaction between sex and grade level among secondary education students.*

## 2. Materials and Methods

### 2.1. Design and Participants

The present study followed a non-experimental, cross-sectional, and descriptive–correlational research design due to its suitability for obtaining descriptive and comparative information from a population at a specific point in time, allowing for the efficient analysis of differences among distinct groups [36]. Sample size was calculated a priori using G*Power 3.1 software [37]. For the MANOVA test (F tests, Global effects), a large effect size was established (f^2^(V) = 0.16), with a significance level of α = 0.05, a statistical power of 0.80, 8 groups (sex × grade level), and 10 variables. The analysis indicated a minimum sample size of n = 203. Accordingly, the convenience sample consisted of 310 students aged between 12 and 16 years (13.9 ± 1.2 years), enrolled across all grades of compulsory secondary education (CSE) in three public secondary schools in Andalusia, Spain. Of the total participants, 58.6% were male (n = 180) and 41.4% were female (n = 130). Regarding grade distribution, 26.4% were enrolled in the 1st year of CSE (n = 82), 23.8% in the 2nd year of CSE (n = 74), 30.9% in the 3rd year of CSE (n = 96), and 17.1% in the 4th year of CSE (n = 53). The inclusion and exclusion criteria for participation were: being enrolled during the current academic year, attending a coeducational school, and providing signed informed consent from parents or legal guardians.

### 2.2. Instruments and Variables

#### 2.2.1. Physical Activity

The Physical Activity Questionnaire for Children (PAQ-C) was used to measure PA. The instrument was validated by Manchola-González et al. [38] and consists of 9 items that assess the frequency of PA performed over the past 7 days, rated on a 1-to-5 Likert scale, with a higher score indicating a higher level of PA. Additionally, the final item asks whether there was any illness or other event that prevented PA during the previous week.

#### 2.2.2. Academic Stress

The Questionnaire on Academic Stress in Secondary Education (QASSE) was used to assess academic stress. This instrument was validated by García-Ros et al. [39] and consists of 24 items that assess the following dimensions: academic overload, interaction with classmates, family pressure, and future prospects, using a Likert-type response scale ranging from 1 (no stress) to 5 (a lot of stress).

#### 2.2.3. Motivation

The Physical Education Motivation Questionnaire (PEMQ) was used to assess motivation. The instrument was validated by Sánchez-Oliva et al. [40] and consists of 20 items that evaluate the following dimensions: intrinsic motivation, identified regulation, introjected regulation, external regulation, and demotivation. A Likert-type response scale ranging from 1 (strongly disagree) to 5 (strongly agree) was used.

The internal consistency of each instrument yielded values greater than α = 0.71 in their respective validations, meaning they are reliable for consistently collecting data. In this study, reliability was analyzed using Cronbach’s alpha, and the results are presented in Table 1.

### 2.3. Procedure

First, a literature review on the study topic was conducted. Subsequently, the research variables were established, and the study objectives were defined. Following this, approval for research involving human participants was requested from the Ethics Committee of the University of Granada, which granted approval under code 3324/CEIH/2023. Afterwards, the management teams of the secondary education schools were contacted and informed about the aims of the study through an information letter. Once authorization from the schools had been obtained, data collection was carried out during March and April 2023 through the in-person administration of paper-based questionnaires during school hours and in the absence of the Physical Education teacher. Participants received the appropriate instructions for questionnaire completion, and members of the research team were present to address any questions. Participant anonymity and data confidentiality were ensured at all times in accordance with the recommendations of the Declaration of Helsinki [41]. Finally, after data collection, corresponding analyses were conducted, leading to the development of the study results, discussion, and conclusions.

### 2.4. Statistical Analysis

For the statistical analysis, IBM SPSS Statistics 28.0 software was used. Initially, descriptive statistics, including mean and standard deviation, were calculated, along with questionnaire reliability using Cronbach’s alpha coefficient. Data normality was assessed using the Kolmogorov–Smirnov test (*p* > 0.05), which led to the use of Spearman’s correlation coefficient for the correlational analyses. For the inferential analyses—MANOVA and ANOVA—by sex (male and female) and grade level (1st, 2nd, 3rd, and 4th year), parametric procedures were retained despite some deviations from statistical assumptions. Previous research has shown that these analyses are generally robust to moderate violations of normality and homogeneity of variances, particularly in studies with moderate-to-large sample sizes [42,43]. Nevertheless, the presence of assumption violations should be acknowledged as a methodological limitation, and the results should therefore be interpreted with appropriate caution. Accordingly, when significant MANOVA results were obtained, univariate analyses (ANOVA) and Games–Howell post hoc tests were subsequently conducted. Effect size was determined using partial eta squared (ηp^2^), interpreted as small (0.02 < ηp^2^ < 0.13), medium (0.13 < ηp^2^ < 0.26), and large (ηp2 > 0.26) [44]. In addition, for the correlational analyses, the magnitude of the coefficients was interpreted as follows: <0.10 trivial, 0.10–0.39 weak, 0.40–0.59 moderate, 0.60–0.79 strong, and 0.80–1.0 very strong [45].

## 3. Results

Table 1 presents the descriptive statistics for PA, academic stress dimensions, and motivation by sex and grade level.

**Table 1:** Descriptive statistics by academic year and sex.

	α	Grade	1st Year of the CSE		2nd Year of the CSE		3rd Year of the CSE		4th Year of CSE		Total	
		Sex	M	SD	M	SD	M	SD	M	SD	M	SD
Physical activity	0.91	Men	2.68	0.87	2.87	0.90	2.77	0.82	3.02	0.70	2.81	0.84
		Women	2.80	0.97	2.72	0.84	2.45	0.66	2.37	1.15	2.58	0.88
		Total	2.72	0.90	2.81	0.87	2.62	0.76	2.75	0.96	2.71	0.86
Academic overload	0.84	Men	3.25	0.86	2.85	0.92	3.11	0.81	3.17	0.97	3.10	0.89
		Women	2.96	0.85	3.39	0.92	3.76	0.74	3.43	1.07	3.43	0.91
		Total	3.14	0.86	3.07	0.95	3.43	0.84	3.28	1.01	3.24	0.91
Interaction with classmates	0.74	Men	2.60	1.07	2.33	0.92	2.16	0.77	2.33	0.77	2.37	0.92
		Women	2.25	0.83	2.66	0.76	2.54	0.83	2.45	1.02	2.48	0.85
		Total	2.47	1.00	2.47	0.87	2.34	0.82	2.38	0.88	2.42	0.89
Family pressure	0.80	Men	2.90	1.16	2.82	1.08	2.67	1.08	2.92	1.20	2.82	1.12
		Women	2.32	1.16	3.38	0.93	3.09	1.19	2.67	0.82	2.90	1.13
		Total	2.69	1.19	3.04	1.05	2.87	1.15	2.82	1.06	2.85	1.12
Future prospects	0.71	Men	2.91	1.09	2.98	1.08	2.80	1.03	2.79	1.10	2.88	1.07
		Women	2.65	1.06	2.98	0.97	3.20	0.92	3.19	0.91	3.02	0.98
		Total	2.82	1.08	2.98	1.03	2.99	0.99	2.96	1.04	2.93	1.03
Intrinsic motivation	0.88	Men	2.84	1.41	3.63	1.07	3.32	1.12	3.75	1.05	3.32	1.24
		Women	3.47	1.17	3.86	0.84	3.02	1.14	3.00	1.08	3.32	1.12
		Total	3.07	1.35	3.72	0.98	3.17	1.14	3.44	1.12	3.32	1.19
Identified regulation	0.84	Men	2.69	1.28	3.48	0.95	2.89	0.97	3.21	1.21	3.03	1.14
		Women	3.42	1.03	3.36	0.92	2.97	1.12	2.72	0.96	3.13	1.05
		Total	2.95	1.24	3.43	0.93	2.93	1.04	3.00	1.13	3.07	1.11
Introjected regulation	0.75	Men	2.61	1.06	3.34	0.97	2.80	0.89	2.82	1.04	2.88	1.02
		Women	2.87	0.97	2.93	0.96	2.72	0.93	2.89	1.05	2.83	0.96
		Total	2.70	1.03	3.17	0.98	2.76	0.90	2.85	1.04	2.86	0.99
External regulation	0.71	Men	2.90	1.14	3.13	1.02	2.72	0.87	2.88	1.06	2.90	1.03
		Women	2.89	1.10	2.93	0.96	2.75	1.05	2.60	0.83	2.80	1.00
		Total	2.90	1.12	3.04	1.00	2.73	0.95	2.76	0.97	2.86	1.02
Demotivation	0.82	Men	2.83	1.30	2.84	1.17	2.09	0.97	2.45	1.30	2.56	1.22
		Women	2.11	1.23	2.39	1.19	2.64	1.07	2.47	1.24	2.43	1.17
		Total	2.57	1.31	2.66	1.19	2.36	1.05	2.46	1.27	2.50	1.20

α = Cronbach’s alpha; M = Mean; SD = Standard Deviation.

Table 2 presents the results of the correlation analysis among the variables using Spearman’s correlation coefficient. Initially, it was observed that PA was positively correlated with intrinsic motivation (*p* < 0.001, rho = 0.301), identified regulation (*p* < 0.001, rho = 0.300), and introjected regulation (*p* = 0.008, rho = 0.151), whereas the relationship with amotivation was negative (*p* = 0.017, rho = −0.135). These correlations showed weak correlation coefficients. On the other hand, the associations among all dimensions of academic stress and among the dimensions of the motivation scale were statistically significant (*p* < 0.001), with moderate-to-strong association coefficients, reflecting the validity of the theoretical construct without indicating a multicollinearity issue.

In the case of amotivation, positive correlations were observed with the academic stress dimensions of academic overload (*p* = 0.028, rho = 0.125), peer interaction (*p* < 0.001, rho = 0.348), family pressure (*p* < 0.001, rho = 0.234), and future perspectives (*p* < 0.001, rho = 0.246). Meanwhile, amotivation was negatively correlated with intrinsic motivation (*p* < 0.001, rho = −0.261) and identified regulation (*p* < 0.001, rho = −0.212), and positively correlated with external regulation (*p* < 0.001, rho = 0.218). Finally, intrinsic motivation was negatively correlated with academic overload (*p* = 0.025, rho = −0.128) and peer interaction (*p* = 0.037, rho = −0.118). External regulation, in turn, was positively associated with peer interaction (*p* = 0.007, rho = 0.154) and future perspectives (*p* = 0.002, rho = 0.174). In addition, a positive correlation was found between introjected regulation and future perspectives (*p* = 0.050, rho = 0.111). All observed correlations fell within the range of weak coefficients.

Regarding the MANOVA, Box’s M test initially showed that the assumption of homogeneity of covariance matrices was not met (*p* < 0.05); therefore, the Games–Howell procedure was adopted. The multivariate tests obtained using Pillai’s Trace indicated a significant effect on the dependent variables according to grade level (F = 1.596, *p* = 0.023, ηp^2^ = 0.051, 1 − β = 0.991), sex (F = 1.976, *p* = 0.036, ηp^2^ = 0.063, 1 − β = 0.874), and the grade level × sex interaction (F = 2.229, *p* < 0.001, ηp^2^ = 0.070, 1 − β = 1.000), with small effect sizes.

As shown in Table 3, the univariate analyses demonstrated that, according to sex, there were statistically significant differences in PA (*p* = 0.016, ηp^2^ = 0.019, 1 − β = 0.677) and academic overload (*p* = 0.006, ηp^2^ = 0.025, 1 − β = 0.794), with higher mean values for males in PA (M = 2.81) and higher mean values for females in academic overload (M = 3.43), both with small effect sizes. Meanwhile, according to grade level, significant differences were found in the variables of academic overload (*p* = 0.040, ηp^2^ = 0.027, 1 − β = 0.672), intrinsic motivation (*p* = 0.005, ηp^2^ = 0.042, 1 − β = 0.873), and identified regulation (*p* = 0.023, ηp^2^ = 0.031, 1 − β = 0.740), also with small effect sizes.

Games–Howell post hoc analyses demonstrated significant differences in the family pressure dimension (between 1st and 2nd year, *p* = 0.044), intrinsic motivation (between 1st and 2nd year, *p* = 0.011, and between 2nd and 3rd year, *p* = 0.008), and identified regulation (between 2nd and 3rd year, *p* = 0.022), with higher mean values observed for 2nd-year students in all cases.

Finally, the Games–Howell test revealed statistically significant values for the sex × grade level interaction. In the case of females, significant differences were found in the variables of academic overload (between 1st and 3rd year, *p* < 0.001), family pressure (between 1st and 2nd year, *p* = 0.001, and between 1st and 3rd year, *p* = 0.018), and intrinsic motivation (between 2nd and 3rd year, *p* = 0.011, and between 2nd and 4th year, *p* = 0.049). Meanwhile, among males, significant values were found for intrinsic motivation (between 1st and 2nd year, *p* = 0.005, and between 1st and 4th year, *p* = 0.003), identified regulation (between 1st and 2nd year, *p* = 0.002, and between 2nd and 3rd year, *p* = 0.046), introjected regulation (between 1st and 2nd year, *p* = 0.002, and between 2nd and 3rd year, *p* = 0.048), and amotivation (between 1st and 3rd year, *p* = 0.009, and between 2nd and 3rd year, *p* = 0.014). Regarding 1st-year students, sex differences were found in the dimensions of family pressure (*p* = 0.020), intrinsic motivation (*p* = 0.016), identified regulation (*p* = 0.003), and amotivation (*p* = 0.007). Among 2nd-year students, significant sex differences were observed in the dimensions of academic overload (*p* = 0.009) and family pressure (*p* = 0.034). In 3rd-year students, sex differences were found in academic overload (*p* < 0.001), peer interaction (*p* = 0.038), and amotivation (*p* = 0.023). Finally, in 4th-year students, statistically significant sex differences were found in PA (*p* = 0.007) and intrinsic motivation (*p* = 0.020).

## 4. Discussion

The purpose of this study was to analyze the relationships among PA, academic stress, and motivation in PE among secondary education students while also determining differences according to sex and grade level. The relevance of this work lies in the joint analysis of these variables, considering that previous studies have demonstrated the benefits that PE can provide in terms of physical and mental health [1,3,5]. These aspects are particularly important in a population facing various academic challenges that may lead to conditions such as academic stress and that require appropriate coping strategies from educational institutions, among which increasing motivation levels [2,35] and promoting regular PA practice [15,17,18,22] are especially noteworthy. Individuals with high motivation tend to be more physically active and overcome difficulties more easily [46,47], in addition to experiencing multiple cognitive, affective, and behavioral benefits that improve student performance [48,49]. Therefore, the findings of this study may provide preliminary information for the design of PE-based educational strategies aimed at promoting students’ psychological and physical well-being. However, given the predominantly weak correlations observed and the small effect sizes identified in the group comparisons, these findings should be interpreted cautiously.

Initially, the correlational analysis showed that PA, although with a low coefficient, was positively associated with the dimensions of motivation characterized by greater self-determination and negatively associated with amotivation. These findings are consistent with previous studies [50,51], which similarly reported these correlations, demonstrating a positive, although weak, association between more self-determined forms of motivation and PA practice. This may be explained by the fact that self-determined motivations are aligned with students’ personal interests and values, providing a greater sense of purpose, self-fulfillment, and overall well-being [52,53]. Furthermore, according to Abdoshahi et al. [54] and Contento & Heredia [55], higher levels of intrinsic motivation and autonomy support are associated with a greater intention to be physically active, which in turn predicts regular PA practice [50]. Conversely, students who experience amotivation tend to perceive PA practice as useless, negatively affecting self-esteem, commitment, and overall life satisfaction [52,53], and contributing to school dropout and disengagement [32,34]. For this reason, teachers are encouraged to promote teaching styles that support autonomy as a means of improving adherence to PA in PE classes [55,56]. However, the small magnitude of the observed associations suggests that motivation represents only one of several factors potentially related to participation in physical activity.

Regarding the dimensions of academic stress, a positive—although weak—correlation was found with amotivation in PE. These results suggest that greater amotivation is associated with lower interest in classroom participation and higher academic stress related to academic overload, family pressure, peer interaction, and future perspectives. This could be explained through Self-Determination Theory by the lack of support and satisfaction of basic psychological needs [31]. According to Ilma et al. [57], from the perspective of Bronfenbrenner’s ecological theory and Self-Determination Theory, the imbalance between academic demands—such as task complexity, workload accumulation, and inefficient instructional design—and students’ perceived lack of competence may generate high levels of stress and reduce intrinsic motivation, even leading to disinterest, disengagement, and a low sense of belonging. Moreover, the perception that efforts are futile reinforces feelings of frustration, limits improvement potential, weakens social connections, and results in low academic engagement [9,53].

In contrast, the findings showed that higher intrinsic motivation was associated with lower academic stress related to academic overload and peer interaction. A possible interpretation is that students who report more self-determined forms of motivation also tend to have stronger interpersonal relationships, as well as greater adaptability and problem-solving ability, which consequently results in better academic performance [3,7,58]. These findings support the notion that academic stress emerges, among other factors, from a reduction in more self-determined motivational sources [49], although the strength of the associations observed in the present study was limited. Therefore, increasing these forms of motivation may be an appropriate way to improve student well-being and increase classroom participation levels. Furthermore, according to Aldridge and McChesney [59], a positive motivational climate acts as a protective factor against the manifestation of inequalities in vulnerable contexts.

The results of the sex-based analyses demonstrated, with small effect sizes, that there were significant differences in PA, with higher levels observed among males. This finding is consistent with previous studies in which males also reported higher PA levels [15,60,61]. Recent reports support this trend, showing that both in Spain and across Europe, females dedicate less time to PA practice [62]. This may be explained, among other factors, by greater involvement in other types of activities, as well as by family and social influences that primarily encourage sports participation among males, in addition to higher rates of sedentary behavior and sports dropout among females [34,63]. Moreover, regular PA practice and opportunities for engagement are mediated by cultural, environmental, and institutional factors [64], and the motives that encourage PA practice differ according to sex [65].

Regarding academic stress related to academic overload, females presented significantly higher stress levels. This finding is consistent with García and González [9], who reported that females experience greater difficulty in managing stress. Similarly, studies by Tharaldsen et al. [14], Hoyt et al. [66], and Marco-Ahulló et al. [67] found higher levels of academic stress among females, associated not only with workload and assignments, but also with the type of tasks requested and the administration of examinations, with worry, anxiety, and difficulty concentrating identified as the most frequent stress triggers. Furthermore, these studies identified that the most effective coping strategies for such stressors were physical exercise among males, and listening to music and watching television among females. Along these lines, Haritay et al. [28] found that academic stress among adolescents is strongly linked to gender, parental education, academic field, and age, and increases directly due to cognitive, emotional, and behavioral factors. Their findings indicated that males experience greater pressure, grade-related anxiety, and self-disappointment, whereas females experience greater workload and exhaustion. Additionally, they argued that these demands translate into divergent behaviors in which factors such as place of residence, family, and academic grade moderate how stress is perceived and experienced, with girls being more vulnerable to high levels of stress. Thus, academic stress appears to be a complex phenomenon associated with multiple sociodemographic and contextual factors. Although some motivational dimensions showed significant associations with academic stress, the weak magnitude of these relationships and the absence of significant correlations between PA and academic stress suggest that their practical contribution should be interpreted cautiously.

The analyses according to grade level demonstrated significant differences, with small effect sizes, in the dimensions of academic overload, intrinsic motivation, and identified regulation. Second-year CSE students presented higher levels of family pressure, intrinsic motivation, and identified regulation compared with first- and third-year students. Furthermore, the sex × grade level interaction proved to be statistically significant, highlighting those females reported higher levels of academic stress as educational level increased. In contrast, among males, more self-determined forms of motivation tended to be significantly higher in the second year, while PA and intrinsic motivation were higher in the fourth year. These findings are consistent with the research conducted by García-Ros et al. [68], in which females similarly reported greater stress related to academic overload in analyses examining grade level and sex × grade interactions, particularly in the final years of CSE, suggesting that females are more vulnerable during the later stages of secondary education. In addition, Haritay et al. [28] found that students in higher grades are more likely to experience academic stress because, as they progress through their schooling, school demands are increasingly perceived—according to the transactional model—as threats that exceed their coping resources.

Likewise, regarding motivation, the findings partially contrast with the study by Cádiz et al. [69], since in that case, grade-level differences were found in the less self-determined dimensions (introjected regulation, external regulation, and amotivation). Nevertheless, they also observed an increasing trend in self-determined motivation in higher grades, particularly among males. This could be explained by the greater tendency to engage in PA and its relationship with high levels of intrinsic motivation [50,56]. However, according to Haritay et al. [28], the contribution of PA appears modest in relation to academic stress when detached from affective and cognitive factors. Thus, promoting PA practice within the context of PE requires strengthening motivation toward more self-determined forms, as these foster autonomy, self-efficacy, perceived competence, and commitment [29,30]. These findings should be interpreted as preliminary evidence to be considered when implementing effective measures aimed at managing and reducing academic stress.

In conclusion, after conducting a cross-sectional analysis of the relationships among PA, academic stress, and motivation toward PE in secondary education, as well as their differences according to sex and grade level, it became evident that academic stress is a complex phenomenon susceptible to various sociodemographic influences, requiring a comprehensive approach to understand its manifestations and, above all, to identify how it may be anticipated, managed, and minimized within the school context. In this regard, fostering self-determined motivation in PE and promoting regular PA practice both within and outside the school environment emerge as highly important pedagogical, curricular, and institutional objectives for improving students’ opportunities for participation and learning in healthy environments that promote physical and mental well-being, particularly in the face of highly prevalent conditions such as academic stress among adolescents [28,35,50].

### Limitations and Future Perspectives

As this is a non-experimental, cross-sectional study, causal relationships could not be established. In addition, the sample may not be representative; therefore, the generalization of the findings to other populations or educational levels should be made with caution. Likewise, the biases associated with self-report measures and the lack of control of confounding variables should be considered. Importantly, although several results reached statistical significance, most of the observed correlation coefficients were weak, and the effect sizes obtained in the MANOVA and ANOVA analyses were predominantly small. Therefore, the practical significance of these findings may be limited and should be interpreted with caution.

These limitations suggest the need for further research using more robust quantitative and qualitative designs that allow for the confirmation and deeper exploration of the findings, as well as the use of measurement instruments (e.g., accelerometers) that may provide more conclusive results regarding students’ perceptions and the influence of motivation in PE and PA practice in relation to the manifestation of academic stress in secondary education. In addition, examining potential mediating and moderating variables may contribute to a more comprehensive understanding of the complex relationships among motivation, physical activity, and academic stress during adolescence.

## 5. Conclusions

In line with the study objectives, the findings suggest that PA is positively associated with more self-determined forms of motivation, although no significant associations were observed between PA and the dimensions of academic stress. Likewise, more self-determined motivational dimensions tended to be associated with lower levels of academic stress, whereas amotivation showed positive associations with several stress dimensions. However, it is important to note that these relationships were generally weak in magnitude. In addition, statistically significant differences according to sex and grade level were identified, although the observed effect sizes were predominantly small, indicating limited practical significance. Therefore, the findings should be interpreted cautiously and understood as preliminary and associative rather than causal evidence. Overall, the results contribute to the understanding of the relationships among PA, motivation, and academic stress in secondary education; however, future longitudinal and experimental studies are required to clarify the direction of these relationships, examine potential causal mechanisms, and determine the practical relevance of these associations over time.

## Figures and Tables

**Table 2:** Correlation analysis.

	2	3	4	5	6	7	8	9	10
1. Physical activity	−0.104	−0.015	0.000	−0.067	0.301 **	0.301 **	0.151 **	0.040	−0.135 *
2. Academic overload		0.352 **	0.468 **	0.486 **	−0.128 *	−0.070	−0.051	−0.005	0.125 *
3. Interaction with classmates			0.442 **	0.517 **	−0.118 *	−0.049	0.056	0.154 **	0.348 **
4. Family pressure				0.449 **	−0.041	0.020	0.013	0.099	0.234 **
5. Future prospects					0.016	0.010	0.111 *	0.174 **	0.246 **
6. Intrinsic motivation						0.703 **	0.464 **	0.294 **	−0.261 **
7. Identified regulation							0.540 **	0.340 **	−0.212 **
8. Introjected regulation								0.569 **	0.050
9. External regulation									0.218 **
10. Demotivation									

* = *p* < 0.05; ** = *p* < 0.01.

**Table 3:** Analysis by grade and sex.

	Grade				Sex			
	F	*p*	ηp^2^	1 − β	F	*p*	ηp^2^	1 − β
Physical activity	0.693	0.557	0.007	0.197	5.892	0.016 *	0.019	0.677
Academic overload	2.801	0.040 *	0.027	0.672	7.778	0.006 *	0.025	0.794
Interaction with classmates	0.400	0.753	0.004	0.129	1.265	0.262	0.004	0.202
Family pressure	2.482	0.061	0.024	0.613	0.083	0.774	0.000	0.059
future prospects	0.816	0.486	0.008	0.226	1.232	0.268	0.004	0.198
Intrinsic motivation	4.414	0.005 *	0.042	0.873	0.128	0.721	0.000	0.065
Identified regulation	3.226	0.023 *	0.031	0.740	0.151	0.698	0.000	0.067
Introjected regulation	2.546	0.056	0.025	0.625	0.113	0.737	0.000	0.063
External regulation	1.364	0.254	0.013	0.362	0.921	0.338	0.003	0.160
Demotivation	0.616	0.605	0.06	0.178	1.134	0.288	0.004	0.186

* = *p* < 0.05; *p =* Significance; ηp^2^ = Partial eta-squared effect size; 1 − β = Statistical power.

## Data Availability

Data are not available due to ethical requirements because they contain personal data.

## Abbreviations

The following abbreviations are used in this manuscript:

PE	Physical Activity
PA	Physical Education
CSE	Compulsory Secondary Education
